# Cognitive Concepts of Craving

**Published:** 1999

**Authors:** Stephen T. Tiffany

**Affiliations:** Stephen T. Tiffany, Ph.D., is a professor in the Department of Psychological Sciences, Purdue University, and a member of the Purdue University Neurosciences Program, West Lafayette, Indiana

**Keywords:** AOD (alcohol and other drug) craving, scientific model, cognition, research method and evaluation, conditioning, expectancy, emotion, memory, AOD use behavior, literature review

## Abstract

Traditional models of craving have been based primarily on the concept of classical conditioning. In recent years, however, researchers increasingly have introduced cognitive concepts, such as memory, expectancies, interpretation, and automatic behavior, into their conceptualizations of craving. These efforts have culminated in the development of four cognitive models of craving: cognitive labeling, outcome expectancy, dual-affect, and cognitive processing. The cognitive processing model posits that although many alcohol use behaviors have become automatized processes in the course of an alcoholic’s drinking career, craving is a nonautomatic process that requires mental effort and is limited by a person’s cognitive capacity. This model also implies that alcohol use and alcohol-seeking behavior can occur in the absence of craving. In addition to introducing various new concepts and models into craving research, the cognitive sciences also offer well-established methodologies for testing these models and analyzing craving processes.

Imagine that you are an alcoholic trying to quit drinking. You have not had a drink in a month, but during the past several days, you have thought about alcohol constantly. These thoughts occupy your mind, making it nearly impossible to concentrate on anything else. Everything around you seems to invoke memories of how pleasant and satisfying drinking can be. You have wrestled with the idea of having a drink, but you have decided to wait at least a little longer. Today, however, after leaving work, you find yourself somewhat mindlessly driving by your favorite bar. You cannot help but notice the front door of the bar propped open, seeming to beckon you inside. You pull over to the curb, park your car, and find yourself standing at the door. As you look through the doorway, it is all so familiar: The bar stools, the television flickering in the corner, and even the smell of stale cigarette smoke are comfortable and inviting. Your heart races and your hands sweat; you realize that this is craving at its worst. You are drawn inexorably into the bar. There is no way you can fight it any longer; you must have a drink.

Although fictional, this situation is not farfetched. In fact, many alcoholics will describe in vivid detail similar stories about craving and relapse (Ludwig 1988). The conventional explanation for this scenario, based on classical conditioning models, is relatively straightforward: Over a long history of drinking, stimuli and events routinely paired with alcohol consumption (e.g., the sight of a bar) become conditioned stimuli—that is, they induce the same responses that are produced by alcohol itself. These conditioned stimuli activate conditioned motivational states^1^ that produce craving experiences, physiological reactions, and alcohol-seeking behaviors. Thus, all the events described in the opening paragraph could be viewed as the consequences of classical conditioning mechanisms (for a review of the classical conditioning model, see sidebar, p. 216, and Tiffany 1995*a*).

**Classical Conditioning and Drug Craving**

**Figure:**
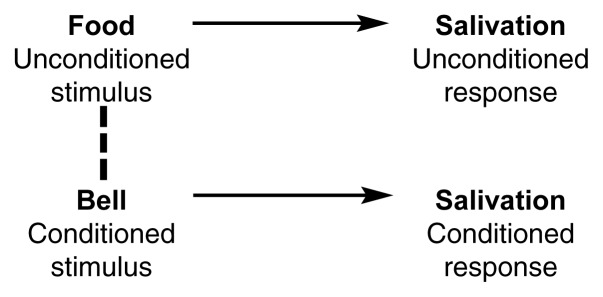
The classical conditioning model. If an unconditioned stimulus (e.g., food) that elicits a certain response is repeatedly paired with a neutral stimulus (e.g., the ringing of a bell), the neutral stimulus eventually becomes a conditioned stimulus that elicits the same response as the unconditioned stimulus.

Russian physiologist Ivan Pavlov (1849–1936) was the first researcher to study the learning procedure called classical conditioning. Pavlov and his coworkers discovered that animals could learn to display certain responses to stimuli that had been paired with other stimuli already eliciting those responses. For example, a dog salivates if food is placed in its mouth. In the terminology of classical conditioning, the food is called an unconditioned stimulus that reflexively elicits an unconditioned response (i.e., salivation) (see figure). If a neutral stimulus (e.g., the sound of a bell) is repeatedly presented at the same time as the unconditioned stimulus (i.e., the food), the neutral stimulus by itself eventually will elicit the same response (i.e., salivation). At that point, the neutral stimulus has become a conditioned stimulus that elicits a conditioned, or learned, response.

The concepts of classical conditioning have had a major impact on theories of alcohol and other drug (AOD) craving. Perhaps the most influential model of conditioned craving was developed by Wikler (1948), who hypothesized that stimuli paired repeatedly with AOD withdrawal could become conditioned stimuli that elicited conditioned withdrawal effects, which, in turn, would generate craving. Addicts experiencing craving would be motivated to seek out and use AODs to relieve the conditioned withdrawal effects. Although this model was originally developed to explain heroin addiction, researchers have also applied it to other drugs, including alcohol (e.g., Ludwig and Wikler 1974). With respect to alcohol craving, a drop in blood alcohol levels would be considered the unconditioned stimulus that activates the unconditioned response (i.e., alcohol withdrawal). Stimuli or situations reliably paired with a decline in blood alcohol levels would become conditioned stimuli. For example, an alcoholic might routinely experience withdrawal in a certain location (e.g., a therapist’s office). After repeated withdrawal episodes (i.e., repeated pairings), seeing or being in this location would be sufficient to trigger conditioned withdrawal reactions and craving.

—Stephen T. Tiffany

ReferencesLudwigAMWiklerA“Craving” and relapse to drinkQuarterly Journal of Studies on Alcoholism3510813019744827273WiklerARecent progress in research on the neurophysiological basis of morphine addictionAmerican Journal of Psychiatry10532933819481889090210.1176/ajp.105.5.329

Numerous components of the scenario described previously, however, go beyond simple conditioning processes. For example, the fictional scene includes descriptions of alcohol-specific *memories*, positive *expectancies* about alcohol use, difficulties in *concentration, decisions* about drinking, *attention* focused on alcohol cues, *interpretations* of physiological reactions, and *automatic behavior* (i.e., automaticity), all of which are cognitive concepts. Craving researchers increasingly apply these concepts in their attempts to understand the processes underlying craving.

What does the term “cognitive approach” mean when applied to craving or to other areas of research? Cognitive approaches investigate the processes that control mental functions, such as communication, learning, classification, knowledge representation, problem-solving, planning, remembering, and decisionmaking. To this end, cognitive science incorporates the contributions of several disciplines, including psychology, philosophy, linguistics, neurobiology, computer science, and engineering. Modern cognitive science generally describes the operation of mental functions in terms of information-processing systems—hypothesized mechanisms that control the acquisition and manipulation of information and translate that information into action.

During the past 25 years, even conventional conditioning models of craving often have invoked cognitive processes. For example, an influential craving theory presented by Ludwig and Wikler (1974) (see sidebar, below) hypothesized that exposure to withdrawal-related cues led to a conditioned withdrawal syndrome, which the alcoholic, through cognitive processes, would experience as craving for alcohol. In another major conditioning model, Wise (1988) described craving as the memory of the positively reinforcing effects of alcohol and other drugs (AODs). Finally, Berridge and Robinson (1995) argued that conditioned drug motivational states were largely unconscious and resulted in conscious experiences of craving only through mechanisms of a process called cognitive interpretation. Although these influential theories all cited cognitive processes as central to the development of craving, they did not elaborate on how those cognitive processes might operate.

Other more recent models describe the role of cognition in craving somewhat more specifically. This article reviews selected cognitive models of craving and discusses their implications for craving research and assessment. Although this review focuses on alcohol craving, neither cognition-based nor conditioning-based contemporary craving models are unique to alcoholism research. Consequently, this review also draws from craving research on other drugs.

## Cognition and Craving Research

The increasing emphasis on cognition in craving research reflects the aftermath of the “cognitive revolution” that swept the social sciences, particularly psychology, in the 1970s. Since then, cognitive perspectives have dominated many of the major fields of psychological research. Even basic models of classical conditioning, which are frequently invoked in craving research, have increasingly incorporated cognitive perspectives (e.g., Rescorla 1988). For example, many modern theories of classical conditioning have adopted an information-processing perspective. This approach posits that the learning of an association between stimuli depends on the extent to which the occurrence of one stimulus provides information about the occurrence of the other stimulus.

When considering the impact of cognitive concepts on craving research, it is useful to distinguish between two types of cognitive theories: cognitive-behavioral models and the cognitive science paradigm. Cognitive-behavioral models (also called social learning models) emphasize such constructs as expectancies, attributions, imitation, and self-efficacy in the control of drinking behavior (e.g., Marlatt and Gordon 1985). These models, which draw heavily from the social and personality psychology of the 1960s and 1970s, are relatively well represented in studies of alcohol dependence. Conversely, the cognitive science paradigm, which focuses on information processing, cognitive architectures, memory, and decision-making, is more clearly representative of contemporary cognitive psychology. Until recently, however, this paradigm did not substantially influence craving research.

Both the cognitive-behavioral and information-processing approaches to craving differ from the conventional conditioning perspectives in several ways. Most important, traditional conditioning models conceptualize craving as a somewhat biologically primal, homogeneous state that directly represents the fundamental motive for AOD use in the addict. Conversely, cognitive approaches consider craving the product of higher order mental functions. Thus, from the cognitive perspective, craving is not a primitive motivational state but a complex, multidimensional process that reflects how AOD-relevant information controls an addict’s behavior. Furthermore, cognitive models contend that craving processes can be measured in ways not envisioned by traditional conceptualizations of craving. (For more information on cognition-based approaches to measuring craving, see the section “Craving and Cognitive Methodology,” p. 222.)

These two perspectives on craving (i.e., cognitive-behavioral and information processing) also have somewhat different implications for the development of interventions to prevent or treat craving. For example, if, as suggested by the conventional view, craving is a biologically primal state, it makes sense to seek biological treatments (e.g., pharmacological therapy) that directly target this motivational state. Conversely, if craving arises from the operation of dynamic, multidimensional information-processing systems, attempts to reduce craving might be most successful if they target the cognitive processes regulating craving.

## Cognitive Models of Craving

Many researchers have speculated that cognition plays an important role in generating craving. To date, however, only four detailed cognitive models of craving exist (see table, p. 218). These include two cognitive-behavioral models—the cognitive labeling model and the outcome expectancy model—and two cognitive science models—the dual-affect model and the cognitive processing model. Each model offers distinct examples of how various cognitive concepts may help explain craving and may therefore complement each other. The four models differ considerably in the extent to which they utilize contemporary cognitive science theories. Indeed, one approach—the cognitive labeling model—uses concepts that are no longer widely accepted in the cognitive sciences. Nevertheless, this model is included here because it still is invoked occasionally when researchers speculate about the cognitive features of craving.

### The Cognitive Labeling Model

One problem associated with conventional conditioning models is explaining how conditioned responses are translated into craving experiences. For example, assume that an alcoholic presented with the sight of his or her favorite bar responds to the situation with a racing heart and sweaty palms. How can these supposed conditioned autonomic responses generate craving? Some craving theorists have suggested that conditioned responses are converted, in some unspecified way, into experiences of craving (e.g., Poulos et al. 1981). Other researchers have hypothesized that craving experiences arise from some form of cognitive interpretation of conditioned reactions (e.g., Ludwig and Wikler 1974; Melchior and Tabakoff 1984). This latter hypothesis has resulted in the formulation of the cognitive labeling model.

The cognitive labeling model of craving is a variant of Schachter and Singer’s (1962) cognition-arousal theory of emotion. According to that theory, an emotional experience results from the interaction between physiological arousal and a cognitive interpretation of the arousal. The interpretation provides an emotional label that determines the quality (e.g., pleasant or unpleasant) of the emotional state. The intensity of that emotion is determined by the extent of the arousal (i.e., the greater the arousal, the more intense is the emotion). As applied to craving, this model implies that alcohol cues (e.g., the sight of a bar) can generate conditioned physiological arousal (e.g., a racing heart). In addition, the cues activate mental processes (e.g., memories of previous drinking occasions) that identify the situation as a setting for drinking. As a result of this cognitive response, the alcoholic interprets the physiological reactions as craving (i.e., provides the reactions with the label “craving”). Stated simply, the alcoholic thinks, “I feel aroused and I am in a situation in which I usually drink alcohol. Therefore, this feeling must be craving.”^2^

Many researchers have speculated that labeling may be crucial to craving experiences (for a review, see Tiffany 1995*b*), although no one has generated a fully developed cognition-arousal theory of craving. At best, cognitive labeling explanations of craving are only loose approximations of Schachter and Singer’s (1962) original theory. Furthermore, these explanations ignore subsequent important modifications of that theory (e.g., London and Nisbett 1974), which have replaced the model’s somewhat vague concept of labeling with a more precise proposal—that the experience of emotion requires causal beliefs or attributions to connect emotional cognitions with arousal.

Any attempt to generate a fully articulated cognition-arousal model of craving, however, may be of little relevance for two reasons. First, the cognition-arousal theory of emotion is generally no longer considered an accepted model of emotional processing (Reisenzein 1983), because research findings have not supported this theory. Second, data from the craving literature suggest that the cognition-arousal model cannot adequately explain all aspects of craving. For example, according to this model, the intensity of craving should be determined by the magnitude of the physiological reaction generated by AOD-related stimuli. In other words, the stronger the physiological reaction to drinking cues is, the stronger the craving generated should be. In general, however, no significant correlations exist between the extent of cue-elicited physiological reactions and the extent of cue-elicited craving (Tiffany 1990).

### The Outcome Expectancy Model

This cognitive model contends that environmental cues can trigger powerful expectations about alcohol’s effects and that those expectations will profoundly influence the alcoholic’s behavior. The expectancies have two essential components: an informational component and a motivational component. The informational component represents specific beliefs about alcohol’s various effects. For example, Marlatt (1985) proposed that alcohol-paired stimuli presented to an alcoholic may generate an expectation or anticipation that alcohol use may produce pleasure, relaxation, or relief from withdrawal. The motivational component of expectancies reflects the desire for experiencing the positive outcome of alcohol consumption. It is as though the alcoholic were thinking, “When I see my friends drinking, I think about how much I enjoy the effects of alcohol, and I want to experience those effects.”

**Table:** Cognitive Models of Craving and Their Major Characteristics

Model	Major Characteristics
Cognitive labeling model	Craving is an emotion generated after exposure to alcohol-related cues that lead to both physical arousal and a cognitive response identifying the arousal as craving; the extent of craving depends on the extent of the arousal.
Outcome expectancy model	Craving is generated after exposure to environmental alcohol-related cues that trigger positive expectations about alcohol’s effects.
Dual-affect model	Craving can be generated by both negative emotional systems (e.g., negative emotional states, aversive events, and withdrawal) and positive emotional systems (e.g., positive emotional states and consumption of small alcohol doses). Positive- and negative-affect craving are mutually exclusive; the extent of craving depends on the extent to which positive- or negative-affect systems are activated.
Cognitive processing model	Craving represents a nonautomatic cognitive process that is activated when the execution of automatized drinking behavior is voluntarily or involuntarily blocked. Craving-inducing situations require cognitive processing and mental efforts and may thereby interfere with other cognitively demanding tasks. Craving is not required for either alcohol seeking or alcohol use.

The outcome expectancy model also distinguishes between “craving” and “urges.” According to this definition, craving is the desire for positive outcomes. This desire triggers urges—that is, the intent to engage in alcohol use. This intent, in turn, precipitates drinking. Thus, craving by itself may not be sufficient for causing alcohol consumption, because a person can desire alcohol without intending to drink.

Since Marlatt’s (1985) development of the original outcome expectancy model, researchers have further elaborated on the cognitive aspects of this theory (e.g., Goldman and Rather 1993). For example, these researchers use several contemporary concepts of memory and information processing to describe the mental processes that control alcohol expectancies. These reformulations have not, however, specifically addressed the craving aspects of Marlatt’s original theory.^3^ Thus, expectancy research generally has focused on the beliefs that people have about alcohol’s effects and on the associations of those beliefs with drinking behavior. Few studies have examined the relationship between craving and situation-specific outcome expectancies.

In Marlatt’s theory, positive outcome expectancies about alcohol should be more readily triggered in some settings than in others. Thus, certain situations that are routinely associated with alcohol consumption, such as being in a bar setting, would be much more likely to activate positive outcome expectancies about the effects of alcohol than, for example, being in church. Furthermore, researchers have not investigated specifically the proposed distinction between craving and urges (Tiffany 1995*b*).

If the outcome expectancy model is correct in assuming that expectancies control alcohol consumption, then a correlation should exist between the extent of the expectancies and the extent of alcohol intake. Indeed, several studies have demonstrated that expectancies about alcohol’s effects tend to correlate significantly with general and situation-specific alcohol consumption levels (Goldman and Rather 1993).

Cooney and colleagues (1987) conducted a study in which they exposed both alcoholics and nonalcoholics to drinking cues and subsequently measured the respondents’ changes in outcome expectancies and craving. The study found some evidence that cue exposure enhanced craving and increased expectancies of positive outcomes in both subject groups. However, the expectancy effects were not found consistently across all measures, and some aspects of the findings could even be interpreted as being inconsistent with the expectancy model (see Tiffany 1995*b*). Nevertheless, to date, too little research exists on expectancies and craving for AODs to allow meaningful conclusions about the validity of the outcome expectancy model.

### The Dual-Affect Model

Baker and colleagues (1987) proposed that craving is controlled by complex emotion-processing systems that influence physiological responses, self-reports of craving and emotion, and drug-seeking behavior. The researchers posited that craving can reflect the operation of both negative and positive emotion, or affect, systems.

Negative-affect craving can be triggered by a negative emotional response or feeling (e.g., depression or anger), aversive events (e.g., having to give a presentation to a group of strangers), AOD withdrawal, cues paired with previous withdrawal episodes, and information that the drug is not available. When activated, the negative-affect system induces craving experiences, drug-seeking behavior, negative affect, and physiological reactions that mimic withdrawal.

Conversely, positive-affect craving is associated with positive emotions and with pleasurable or positively reinforcing AOD effects. This craving system is activated by positive emotional states, a small AOD dose, cues paired with AOD use, and information that the drug is available. Activation of this system induces craving, drug-seeking behavior, positive affect, and physiological responses that mimic the stimulating AOD effects.

An important feature of the dual-affect model is that both positive- and negative-affect craving systems are thought to be mutually inhibitory—that is, stimulation of one system would suppress activation of the other system. Consequently, the model assumes that a person cannot experience both positive- and negative-affect craving simultaneously.

According to the dual-affect model, craving systems are structured at a cognitive level into propositional networks—memory networks that store information on the stimuli that trigger the craving system, AOD-related responses, and the meaning or interpretation of stimuli and responses. A given network is activated when environmental stimuli match the stimulus information stored in that network.

The extent of the activation (and, consequently, craving) depends on the extent to which multiple cues match the triggering stimuli stored in the propositional network. For example, an alcoholic in a positive, celebratory mood may experience some alcohol craving. The same alcoholic also may experience craving when presented with the opportunity to consume an alcoholic beverage. The intensity of the craving, however, may differ in each situation. Furthermore, the person’s craving will be considerably stronger if he or she is in a positive mood and is given the opportunity to drink.

The activation level of a network also determines the consistency of the various responses generated by that network. That is, as a network becomes more fully engaged, stronger correlations should exist between the extent of self-reported craving and the extent of drug-seeking behavior. Finally, the dual-affect model predicts that partial activation of a propositional network should lower the threshold for further activation of that network. For example, an alcoholic in a celebratory mood (i.e., whose craving system is partially activated) should respond more strongly to the presentation of alcohol cues than would an alcoholic in a neutral mood (i.e., whose craving system is not activated).

Unlike other cognitive models of craving, the dual-affect model has been developed sufficiently to allow researchers to generate and test fairly precise predictions. For example, the model predicts that negative affect and stress should trigger craving more effectively in drug-deprived than in non-drug-deprived addicts. Furthermore, the model proposes that positive- and negative-affect craving cannot be activated simultaneously. These propositions can readily be tested. Moreover, investigators can easily assess predictions on whether partial activation of one network lowers the threshold at which the person responds to other relevant stimuli.

Several studies have provided partial evidence for some of the model’s predictions. For example, Zinser and colleagues (1992) found that negative affect correlated with craving levels in smokers undergoing nicotine withdrawal but not in continuing smokers. Certain other predictions, however, have not been well supported (Tiffany 1995*b*). Most important, only limited evidence suggests that positive- and negative-affect craving are mutually exclusive (Maude-Griffin and Tiffany 1996; Tiffany and Drobes 1991). In addition, research with cigarette smokers has not yielded any evidence that deprived smokers are specifically sensitized to smoking cues (Maude-Griffin and Tiffany 1996; Drobes and Tiffany 1997). The available evidence therefore suggests that craving networks may not be organized as described by the dual-affect model. Most of those studies, however, have been conducted with cigarette smokers. Almost no research has applied the model specifically to alcohol craving.

### The Cognitive Processing Model

The models previously described assume that craving and AOD use are tightly coupled—in other words, that craving is at the motivational core of AOD use in the addict. Conversely, the cognitive processing model of AOD use and craving proposes that the regulation of AOD use in experienced addicts can function independently of the processes that control craving (Tiffany 1990). According to the cognitive processing model, addictive AOD use is regulated by what cognitive psychologists refer to as automatic processes, whereas craving represents the operation of nonautomatic processes.

What is an automatic process? Many activities are controlled by cognitive processes that operate quickly and effortlessly and require little focused attention. For example, people generally eat, groom, dress, walk, drive, talk, and read while paying little or no attention to exactly how they perform these functions. In fact, many daily activities have become so automatic that people may have difficulty remembering what their performance was like when they first acquired those skills. In contrast, when learning a new skill, its execution requires considerable effort and focused attention. With practice, however, performance improves, and what once was a demanding and clumsy activity becomes effortless and highly coordinated. According to cognitive psychologists, this kind of transformation marks the transition from nonautomatic to automatic functioning (Shiffrin and Schneider 1977).

The cognitive processing model of craving proposes that over a long history of drinking, many of the actions involved in acquiring and consuming alcohol become automatized for alcoholics (see figure below). For example, following years of drinking at a particular bar every day after work, an alcoholic may unthinkingly walk into the bar even though he or she has resolved to quit drinking. Consequently, the actions of a highly practiced addict, during both regular use and relapse, may be viewed not as a consequence of craving but as an example of the behaviors exhibited during the execution of any automatized skill (Tiffany and Carter 1998). As with any activity that is performed repeatedly, alcohol consumption by an alcoholic can be seen as readily triggered by certain stimuli—stereotyped, effortless, difficult to control, and regulated largely outside of awareness. Ludwig (1988, p. 92) provided an excellent description of automatic drinking:

**Figure:**
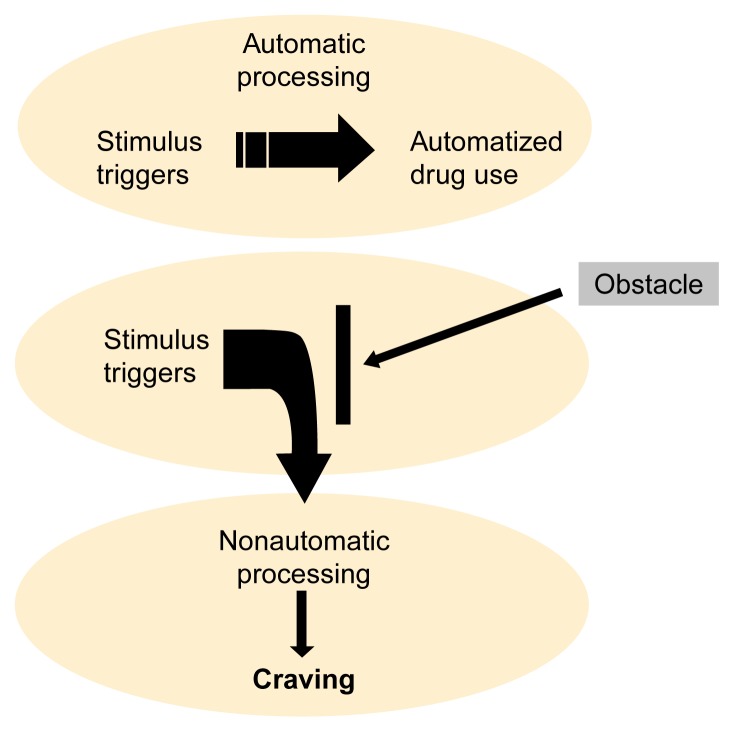
The cognitive processing model. In an alcoholic who is not trying to quit drinking, alcohol use is controlled by automatic cognitive processes. Under these circumstances, “stimulus triggers” activate automatic processes that result in automatized drug use, and craving plays no role in the control of drinking. When the automatized alcohol-use sequences (e.g., driving to a favorite bar, entering, sitting down at the bar, and ordering a drink) are blocked by an environmental obstacle (e.g., the bar is closed), the alcoholic must activate nonautomatic processes to cope with that problem. These nonautomatic processes generate craving for alcohol.

“Others essentially think instinctively, short circuiting both imagery and cognitions, and are inclined to act without knowing why. When alcohol becomes readily available, they drink before they think.”

In contrast to automatic processing, nonautomatic processing can be characterized as slow, flexible, intention dependent, requiring cognitive effort, and restricted by limited cognitive capacity. Nonautomatic processes occur under three kinds of circumstances: (1) when a person first learns a skill, (2) when a highly automatized sequence is activated but some environmental obstacle blocks the completion of that sequence, and (3) when a person wants to prevent the execution of activated automatized sequences (Shiffrin and Schneider 1977). The cognitive processing model hypothesizes that craving represents nonautomatic processes that are activated simultaneously with automatized AOD use sequences. The nonautomatic processes are activated either to overcome obstacles to successfully completing automatized AOD use or to prevent the execution of an automatized sequence. These two situations generate, respectively, the craving observed in alcoholics who are not attempting to quit drinking and the craving observed in alcoholics who are attempting abstinence.

The first scenario is exemplified by the alcoholic who goes to his or her favorite bar and discovers that it is closed for remodeling. Resolution of this unexpected inconvenience requires nonautomatic processes, causing changes in overt behavior, craving and emotional distress, and physiological responses. For example, the alcoholic will take action to overcome the obstacle (e.g., drive to another bar). In addition, the alcoholic will express desire for alcohol, intentions to find and consume alcohol, and frustration and anger at being thwarted in his or her attempt to drink. The alcoholic also will likely display physiological responses (e.g., changes in heart rate or sweat gland activity) that reflect either the physical or cognitive demands of the craving situation.

The second scenario represents the situation confronting alcoholics who are trying to quit drinking. Every day they are faced with cues and situations that trigger portions of their automatic drinking behaviors; consequently, they must exert considerable mental effort to keep themselves from drinking. The constant battles with craving may leave the abstinent alcoholic mentally exhausted and unable to cope effectively with the cognitive demands of everyday life. The cognitive effort associated with abstinence may be sustainable as long as no additional problems arise (e.g., in the workplace or in relationships with family and friends). In stressful, challenging situations (e.g., when faced with a tight deadline at work), however, the abstinent alcoholic may find it easier to succumb to alcohol craving and take a drink rather than struggle with both craving and work-related pressure.

Overt behavior, self-reports of craving and emotional distress, and autonomic physiological responses are typical reactions that result from the activation of craving processes. The cognitive processing model, however, proposes that craving may influence a fourth category of responses not typically addressed in craving studies—that is, responses to the cognitive demands of craving.

According to the model, a craving-inducing situation presents a problem that demands a solution. Furthermore, the model posits that solving this problem requires nonautomatic cognitive processes that function at the expense of other activities that also necessitate nonautomatic processing. Because nonautomatic processes involve mental effort that exhausts limited cognitive resources, the alcoholic trying to solve craving-associated problems will have little mental capacity left for coping with other cognitively demanding situations. Thus, the nonautomatic cognitive demands of craving processes can account for the disruptive impact of craving on daily functioning.

The cognitive processing model allows for several explicit predictions about the cognitive organization of craving and AOD use, the behavioral manifestations of craving, and the role of automatic and nonautomatic processes in relapse. Many of these predictions have not yet been evaluated systematically; however, various studies have strongly supported the core assumption of this model—that craving is not necessary for drug seeking or AOD use (Tiffany 1990, Tiffany and Carter 1998; Tiffany and Conklin in press). Neither studies of craving and alcohol consumption in the laboratory nor studies of craving and relapse in the real world have provided strong evidence that craving is directly responsible for alcohol use in alcoholics (Tiffany and Conklin in press*)*. For example, the alcohol amounts consumed by alcoholics in laboratory studies are only weakly correlated with the alcoholics’ descriptions of craving. Moreover, studies of relapse episodes indicate that addicts rarely identify craving as a major direct trigger for resuming AOD use (Tiffany and Carter 1998).

Numerous studies also have supported the cognitive processing model’s prediction that craving activation should disrupt cognitive functioning (Sayette in press). For example, Sayette and colleagues (1994) exposed people receiving inpatient alcoholism treatment to both alcohol-related cues (e.g., a glass of their favorite alcoholic beverage) and neutral cues (e.g., a glass of water). During cue exposure, the subjects were asked to press a button whenever they heard a brief sound, and their reaction times were recorded. The study found that reaction times were slower in the presence of alcohol-related cues than in the presence of neutral cues, suggesting that the alcoholics’ ability to perform the button-pressing task was disrupted by the cognitive processing triggered by the alcohol cues. These findings support the cognitive processing model’s hypothesis that craving is cognitively disruptive and therefore exerts a toll on other cognitively demanding tasks.

Beyond their implications for the cognitive processing model, this and similar studies demonstrate how the cognitively intrusive and disruptive features of craving—features that addicts so often complain about—can be studied under controlled laboratory conditions. More generally, the studies illustrate the usefulness of measures and methods derived from cognitive sciences for investigating craving processes. These methods are discussed more fully in the following section.

## Craving and Cognitive Methodology

The cognitive sciences bring not only a fresh perspective to the conceptualization of craving but also a variety of well-established procedures and measures for studying craving processes. This cognitive methodology has considerable potential for enriching the traditional assessment approaches generally used in craving studies. Most laboratory studies of craving expose addicts to AOD-related cues and then measure self-reported craving; autonomic reactions (e.g., heart rate); and, occasionally, AOD seeking or use. In those studies, alcoholics confronted with alcohol-related cues typically exhibit increases in self-reported craving, heart rate, sweat gland activity, and salivation (Carter and Tiffany in press; Tiffany et al. in press). Thus, those studies demonstrate (for alcohol as well as for other drugs) that both nonautomatic processes, such as craving, and autonomic reactions can be manipulated in the laboratory (Carter and Tiffany in press). Nevertheless, such analyses are unlikely to reveal much about the dynamics of the psychological mechanisms that regulate craving.

Cognitive scientists have developed highly sophisticated tasks and measures to dissect the operation of complex cognitive processes. One technique, the dual-task procedure, has been used in several investigations into the cognitive demands of craving (Tiffany 1990) and is a well-established approach for investigating the extent to which the processing demands of one task interfere with performance of a second task (Sayette in press). In craving research, the primary task is a procedure designed to activate the nonautomatic processes that generate craving. For example, smokers might be asked to imagine a situation in which they have a strong desire to smoke (Cepeda-Benito and Tiffany 1996). The secondary, or probe, task generally is a simple reaction-time task in which subjects are asked, for example, to generate a response (e.g., press a button) whenever they perceive a probe stimulus (e.g., hear a tone). The investigator then measures the subject’s reaction time in response to the stimulus. Slowed reaction times during craving are assumed to reflect the processing demands of the craving task. This approach allows for the quantification of the old adage that it is hard to do two things at once, particularly if one of the things (i.e., coping with craving) is especially difficult.

As noted earlier in this article, many studies have demonstrated that craving induction can disrupt performance of a secondary task. These studies also suggest, however, that reaction-time measures and craving reports may not be controlled entirely by the same underlying psychological processes. For example, some analyses determined only weak or even statistically insignificant correlations between slowed reaction times and craving levels (e.g., Cepeda-Benito and Tiffany 1996). This observation is similar to the finding that craving levels do not correlate strongly with physiological responses or measures of AOD use. The weak relationships among self-reported craving, cognitive measures, physiological reactions, and assessments of AOD use underscore the growing recognition among craving researchers that responses elicited in craving situations reflect the combined effects of multiple psychological processes.

Although initial studies of craving and cognitive processing have yielded promising results, these studies represent only a small part of the potential of cognitive technology for revealing the complexities of craving processes (Sayette in press). Thus, the studies have produced additional questions that remain unanswered, including which aspect of craving disrupts processing or depletes cognitive resources. Several potential explanations exist for this observation, including the following:

- AOD-related stimuli may be so “attention grabbing” that they prevent addicts from focusing on a secondary task.
- AOD-related cues may induce some degree of general arousal, thereby interfering with the addict’s ability to complete the secondary task.
- AOD-related cues may conjure up upsetting memories about alcohol, and those negative recollections may reduce the addict’s motivation to perform.
- Craving may prime motor systems organized to seek and use drugs, and the activation of those systems may conflict with the motor demands of the button-pressing task.
- Craving may reflect a problem-solving activity that exhausts resources that might otherwise be devoted to responding quickly on the secondary task.

Cognitive science offers many refined techniques for identifying the source(s) of disruption in dual-task procedures (e.g., Wickens 1984). For example, craving researchers can use secondary tasks that vary systematically along several critical dimensions (e.g., motor demands, cognitive complexity, and ease of detecting the probe stimulus). Cognitive scientists have repeatedly used systematic manipulations of these dimensions to analyze the processing demands of cognitive tasks. The results of similarly designed craving studies could offer fascinating insights into the cognitive dynamics of craving processes. Moreover, such research could provide a model of how the conceptual and methodological contributions of cognitive sciences could be exploited to discover how alcoholics and other addicts process information about AODs.

In addition to providing techniques for assessing cognitive aspects of craving, cognitive sciences offer immense potential for craving research, because modern theories of cognitive processing often are so well developed that they can be used to generate mathematical or computer models of mental functioning. Such models offer a level of predictive precision and testability that cannot be achieved with less formally developed theories. To date, theories of craving or of addictive behavior in general have not been developed sufficiently to allow for the generation of formal mathematical or computer models. The cognitive sciences, however, offer a standard of theorizing that if fully adopted by craving researchers, would represent a quantum leap in the scientific study of craving processes.

## Cognition and the Future of Craving Research

The cognitive models discussed in this article offer perspectives on craving processes that differ markedly from conventional, conditioning-based concepts of craving. In some cases, such as the cognitive labeling hypothesis, the models merely place a cognitive spin on basic conditioning approaches. Conversely, some theories, such as the cognitive processing model, depart radically from traditional assumptions about the organization and function of craving processes. Despite their differences, however, cognitive approaches share common themes. Most important, these approaches posit that craving arises from the operation of information-processing systems. In other words, these models view craving not merely as a primitive drive state but as the product of higher order cognitive functions.

Cognitive models offer researchers the opportunity to study craving processes with measures that extend far beyond the standard inventory of variables suggested by conventional craving theories. For example, studies of attributions, outcome expectancies, intentions, memory organization, and performance-based indicators of mental functions likely will elucidate many of the mechanisms underlying craving. Although cognitive approaches to craving have considerable potential, they still have shortcomings. For example, many of the predictions generated by cognitive models have not been systematically evaluated. Moreover, some of the models are insufficiently developed or invoke constructs that no longer represent contemporary research and theory in the cognitive sciences.

Despite these problems, the presence of cognitive concepts, measures, and methods in craving research will almost inevitably increase. This assertion is based at least in part on the observation that the theories of the behavioral sciences in general increasingly incorporate cognitive dimensions. Indeed, the cognitive perspective has become the dominant paradigm in most behavioral sciences. Craving research has always tracked—albeit with some delay—major trends in the behavioral sciences. Beyond this “inevitability of history,” however, cognitive approaches will be welcomed into studies of alcohol craving because of their potential for helping researchers disentangle complex craving data. For example, over the next few years, scientists will face entirely new types of results generated by brain imaging studies of craving. To understand the neurobiological correlates of craving identified by such studies and to identify the association between neurobiological features and the psychological characteristics of craving, alcohol researchers almost certainly will need to apply state-of-the-art cognitive processing models and methods.

## Footnotes

1 For a definition of this and other technical terms used in this article, see glossary, p. 223.

2 Some emotion theorists have hypothesized that such thought processes probably occur unconsciously in the everyday experience of emotion.

3 Indeed, in their elaboration of expectancy theory, Goldman and Rather (1993) have adopted the position suggested by Tiffany (1990) that the motivational control of alcohol use in the highly practiced alcoholic may be automatized and not reflected in reports of craving (also see section “The Cognitive Processing Model,” p. 220).

## GLOSSARY

Attributions: Inferences on the causes and effects of behavior.
Autonomic responses: Physiological responses that are controlled by the autonomic nervous system (e.g., heart rate, blood pressure, and sweat gland activity). These responses are not under conscious control.
Conditioned withdrawal syndrome: The experience of physiological symptoms (e.g., sweating, tremors, and anxiety) of withdrawal from alcohol and other drugs that is not caused by the actual withholding of the drug but by stimuli that have been associated with previous withdrawal episodes.
Expectancies: Expectations regarding the effects of a behavior or a drug.
Motivational state: A psychological state that initiates or modifies a certain behavior (e.g., seeking and consuming alcohol or other drugs).
Paradigm: A set of related theories or models.
Positively reinforcing effects: The effects of a drug (e.g., alcohol) that increase the probability that a person will attempt to consume more of that drug.
